# Long use of continuous positive airway pressure protects against the development of treatment-requiring retinopathy of prematurity

**DOI:** 10.1038/s41598-022-11509-w

**Published:** 2022-05-12

**Authors:** Shutaro Suga, Yuki Kyono, Takumi Kido, Ruka Nakasone, Shinya Abe, Mariko Ashina, Kandai Nozu, Kazumichi Fujioka

**Affiliations:** 1 Department of Pediatrics, Kobe University Graduate School of Medicine, 7-5-2, Kusunoki-cho, Chuo-ku, Kobe, 650-0017 Japan; 2 Department of Pediatrics, School of Medicine, University of Occupational and Environmental Health, Japan, Kitakyushu, Japan

**Keywords:** Risk factors, Epidemiology, Paediatric research, Eye diseases

## Abstract

Although preterm infant mortality is low, the proportion of patients with treatment-requiring retinopathy of prematurity (TR-ROP) is high in Japan. Various multicenter studies have reported the risk factors for TR-ROP; however, no large-scale studies have been conducted in Japan. We retrospectively analyzed 13,645 infants born at < 28 weeks’ gestation (January 1, 2009–December 31, 2018), and registered in the Neonatal Research Network of Japan database. TR-ROP was defined as ROP requiring retinal laser photocoagulation and/or intravitreal anti-vasoendothelial growth factor drugs. Multivariable logistic regression analysis was performed to identify factors associated with TR-ROP development. The median gestational age of enrolled infants was 26 weeks (interquartile range [IQR], 24–27 weeks), median birth weight was 760 g (IQR, 620–918 g). Proportion of patients with TR-ROP was 30.3%. TR-ROP was significantly associated with birth at < 26 weeks’ gestational age (adjusted odds ratio [aOR] 1.54), blood transfusion (aOR 1.49), invasive ventilation ≥ 28 days (aOR 1.41), sepsis (aOR 1.29), birth weight < 750 g (aOR 1.28), intraventricular hemorrhage (aOR 1.33), delayed achievement of full enteral feeding > 14 days (aOR 1.28), and continuous positive airway pressure (CPAP) therapy ≥ 28 days (aOR 0.79). Supplemental oxygen ≥ 28 days was not associated with TR-ROP development. Lower gestational age at birth and birth weight, blood transfusion, prolonged invasive ventilation, sepsis, intraventricular hemorrhage, and delayed achievement of full enteral feeding were risk factors for TR-ROP, whereas CPAP use was protective against TR-ROP.

## Introduction

Retinopathy of prematurity (ROP) is a major neonatal morbidity and the most common avoidable cause of childhood blindness in middle-income countries^1^. In 2010, approximately 170,000 preterm infants developed ROP, with 54,000 of them requiring treatment worldwide^2^. According to North American statistics, 89.4% (1202/1344) of preterm infants born at ≤ 28 weeks of gestation and weighing ≤ 750 g developed ROP in at least one eye. By comparison, only 6.3% (33/529) of preterm infants born at ≥ 30 weeks of gestation with a birth weight of ≥ 1500 g developed ROP^3^. Therefore, among preterm infants, those born at an extremely low gestational age (before 28 weeks of gestation) are at a higher risk of developing ROP. Regarding the prevalence of treatment-requiring ROP (TR-ROP), the International Network for Evaluating Outcomes (iNeo) reported that 24.9% of preterm infants born between 24 and 28 weeks of gestation (2007–2013) required treatment for ROP. In their report, Japan had the highest survival rate (92%) but also the highest proportion of patients with TR-ROP (30.4%) among the participating countries^4^. Notably, a British nationwide population-based study reported that the proportion of patients with TR-ROP was 2.5 times higher in the recent years, than previously estimated^5^. The steady increase in the proportion of patients with TR-ROP is thought to be due to the increase in the survival rate of extremely preterm infants^6^.

Although genetic predisposition is thought to be involved in ROP^7^, the major monogenic mutations involved in the onset and severity of ROP have not been identified. In the United States, severe ROP has been reported to occur more frequently in Caucasian than in African-American infants, suggesting the presence of racial risk variation in ROP^8^. However, this racial difference might be explained by racial variation in socioeconomic status^9^. Until now, various multicenter studies have been conducted worldwide, including in the United States^10,11^ and Europe^12,13^, on the risk factors and treatment of ROP. However, few large-scale studies have been conducted in the Asian population. The main risk factors of ROP include the two main risk factors of prematurity (lower gestational age and birth weight), and oxygen supplementation^14,15^; several other factors such as intraventricular hemorrhage (IVH), sepsis, necrotizing enterocolitis (NEC), respiratory distress syndrome (RDS), blood transfusion, mechanical ventilation, failure to gain weight, treatment-requiring patent ductus arteriosus (PDA), thrombocytopenia^16^, antenatal corticosteroid^17^, and erythropoietin therapy^9^, have also been reported. Regarding the risk of oxygen supplementation, several reports have stated that the proportion of patients with ROP and its treatment rates have decreased significantly since the introduction of lower oxygen saturation targets in the acute phase owing to the introduction of pulse oximetry in the neonatal intensive care unit (NICU)^18^. In the findings of the NeOProM study, a meta-analysis of all the trials, low oxygen targets (85–89%) were associated with an increased incidence of necrotizing enterocolitis and death, but a reduced proportion of patients with TR-ROP^19^. While neonatal management that refrains from excessive oxygen use has become established, there is no large-scale study examining the relationship between oxygen supplementation and ROP in Japan.

In this study, risk factors for TR-ROP in Japan between 2009 and 2018 were investigated using the Neonatal Research Network of Japan (NRNJ) database. This is a prospective database consisting of 218 institutions that register the clinical information of newborns weighing ≤ 1500 g at birth or of those born at a gestational age of < 32 weeks.

## Results

A total of 18,160 newborns were born at 22–27 weeks of gestation and/or had a birth weight of < 1500 g, between January 1, 2009 and December 31, 2018. Of these infants, 6617 were excluded due to the following reasons: death before discharge (2000 infants), major congenital anomalies (736 infants), transfer to other hospitals (1419 infants), and insufficient data regarding the diagnosis of ROP (2462 infants). Among the remaining 13,645 infants, 7041 (51.6%) eventually developed ROP with 4136 (30.3%) requiring treatment (TR-ROP) (Supplementary Fig. S1). The trends in the proportion of patients with TR-ROP and non-TR-ROP did not change significantly through the study period (Supplementary Fig. S2). There was a slight but significant decreasing trend in the gestational age of newborns screened for ROP (*p* < 0.01, Supplementary Fig. S3).

The clinical characteristics of infants with ROP are shown in Table 1. These infants were born at a median gestational age of 26 weeks (IQR, 24–27 weeks) and had a median birth weight of 760 g (IQR, 620–918 g). Overall, the proportion of infants with TR-ROP was 30.3%. Comparing the characteristics of infants in the TR-ROP and non-TR-ROP (n = 9509) groups, gestational age [TR-ROP vs. non-TR-ROP: 25 (24–26) vs. 26 (25–27) weeks], birth weight [674 (565–814) vs. 806 (658–954) g], and Apgar scores at 5 min [6 (5–8) vs. 7 (6–8)] were significantly lower, multiple births (18.0 vs. 16.2%) was significantly more common in the TR-ROP group (all *p* < 0.01). Regarding morbidities, the proportion of patients with RDS (84.1 vs. 78.8%), sepsis (18.2 vs. 10.3%), NEC (3.0 vs. 1.6%), IVH (31.2 vs. 19.1%), and treated PDA (60.5 vs. 55.9%) were significantly higher in the TR-ROP group (all *p* < 0.01). Regarding neonatal management, blood transfusion (80.6 vs. 58.0%), erythropoietin for anemia (93.8 vs. 92.3%), invasive ventilation (46 vs. 30 days), supplemental oxygen (85 vs. 66 days), oxygen use at 36 weeks of corrected age (68.5 vs. 64.6%), and parenteral nutrition (93.8 vs. 91.4%) were significantly more common, time from birth to full enteral feeding (15 vs. 13 weeks) was significantly longer, and use of CPAP (29 vs. 32 days) was significantly lower in the TR-ROP group compared to the non-TR-ROP group.

**Table 1:** Clinical characteristics of enrolled infants.

	TR-ROP n = 4136	Non-TR-ROP n = 9509	*P* value
Gestational age, weeks	25 (24–26)	26 (25–27)	< 0.01
Birth weight, g	674 (565–814)	806 (658–954)	< 0.01
Multiple births	744/4136 (18.0)	1484/9157 (16.2)	< 0.01
Male sex	2210/4135 (53.4)	4878/9155 (53.3)	0.86
Apgar Score 5 min	6 (5–8)	7 (6–8)	< 0.01
**Morbidities**			
RDS	3466/4122 (84.1)	7181/9117 (78.8)	< 0.01
Sepsis	747/4110 (18.2)	937/9096 (10.3)	< 0.01
NEC	124/4125 (3.0)	149/9123 (1.6)	< 0.01
IVH	1283/4118 (31.2)	1741/9114 (19.1)	< 0.01
Treated PDA	2498/4127 (60.5)	5003/9107 (55.9)	< 0.01
**Neonatal managements**			
Blood transfusion	3115/4113 (80.6)	5277/9096 (58.0)	< 0.01
Erythropoietin use for anemia	3852/4107 (93.8)	8384/9081 (92.3)	< 0.01
Invasive ventilation, days	46 (30–66)	30 (12–48)	< 0.01
CPAP, days	29 (14–44)	32 (19–45)	< 0.01
Supplemental oxygen, days	85 (59–127)	66 (42–101)	< 0.01
Oxygen use at 36 weeks of corrected age	2253/3289 (68.5)	5812/9012 (64.6)	< 0.01
Duration from birth to full enteral feeding	15 (11–23)	13 (10–8)	< 0.01
Parenteral nutrition	3870/4127 (93.8)	8341/9124 (91.4)	< 0.01

*ROP* retinopathy of prematurity, *TR-ROP* treatment-requiring retinopathy of prematurity, *RDS* respiratory distress syndrome, *NEC* necrotizing enterocolitis, *IVH* intraventricular hemorrhage, *PDA* patent ductus arteriosus, *CPAP* continuous positive airway pressure.

To determine the risk factors of TR-ROP, we performed multivariable logistic regression analysis (Table 2). TR-ROP was significantly associated with birth at a gestational age of < 26 weeks (adjusted odds ratio [aOR] 1.80, 99% CI 1.58–20.5), blood transfusion (aOR 1.57, 99% CI 1.37–1.79), invasive ventilation ≥ 28 days (aOR 1.50, 99% CI 1.28–1.70), sepsis (aOR 1.35, 99% CI 1.16–1.57), birth weight < 750 g (aOR 1.46, 99% CI 1.30–1.66), IVH (aOR 1.33, 99% CI 1.18–1.50), delayed achievement of full enteral feeding > 14 days (aOR 1.28, 99% CI 1.15–1.43), and CPAP ≥ 28 days (aOR 0.84, 99% CI 0.75–0.94) (all *p* < 0.001). Supplemental oxygen ≥ 28 days was not associated with the development of TR-ROP.

**Table 2:** Multivariable logistic regression analysis.

Variables	TR-ROP n = 4136	Non-TR-ROP n = 9509	Crude OR (99% CI)	*P* value	Adjusted OR (99% CI)	*P* value
Gestational age < 26 weeks	2814/4136 (68.0)	3608/9157 (39.4)	3.22 (2.91–3.56)	< 0.001	1.80 (1.58–2.05)	< 0.001
Blood transfusion	3315/4113 (80.6)	5277/9096 (58.0)	2.98 (2.67–3.33)	< 0.001	1.57 (1.37–1.79)	< 0.001
Invasive ventilation ≥ 28 days	3191/4044 (78.9)	4887/8996 (54.3)	3.09 (2.77–3.45)	< 0.001	1.50 (1.28–1.70)	< 0.001
Sepsis	747/4110 (18.2)	937/9096 (10.3)	1.89 (1.66–2.17)	< 0.001	1.35 (1.16–1.57)	< 0.001
Birth weight < 750 g	2678/4132 (64.8)	3652/9139 (40.0)	2.74 (2.49–3.02)	< 0.001	1.46 (1.30–1.66)	< 0.001
IVH	1283/4118 (31.2)	1741/9114 (19.1)	1.87 (1.68–2.09)	< 0.001	1.33 (1.18–1.50)	< 0.001
Delayed achievement of full enteral feeding (> 14 days)	2170/3928 (55.2)	3653/8708 (41.9)	1.72 (1.56–1.89)	< 0.001	1.28 (1.15–1.43)	< 0.001
Erythropoietin use for anemia	3852/4107 (93.8)	8384/9081 (92.3)	1.18 (0.98–1.42)	0.02	0.85 (0.68–1.05)	0.05
NEC	124/4125 (3.0)	149/9123 (1.6)	1.86 (1.36–2.54)	< 0.001	1.19 (0.84–1.69)	0.20
Parenteral nutrition	3870/4127 (93.8)	8341/9124 (91.4)	1.41 (1.17–1.70)	< 0.001	1.00 (0.81–1.26)	0.96
Supplemental oxygen ≥ 28 days	3746/4097 (91.4)	7694/9073 (84.8)	1.84 (1.58–2.15)	< 0.001	1.00 (0.84–1.20)	0.96
RDS	3466/4122 (84.1)	7181/9117 (78.8)	1.39 (1.23–1.57)	< 0.001	1.03 (0.89–1.18)	0.62
CPAP ≥ 28 days	2129/4049 (52.6)	5388/9005 (59.8)	0.75 (0.68–0.82)	< 0.001	0.84 (0.75–0.94)	< 0.001

*ROP* retinopathy of prematurity, *TR-ROP* treatment-requiring retinopathy of prematurity, *OR* odds ratio, *RDS* respiratory distress syndrome, *NEC* necrotizing enterocolitis, *IVH* intraventricular hemorrhage, *CPAP* continuous positive airway pressure.

Adjusted values were obtained from a logistic regression analysis after adjusting for male sex, multiple births, Apgar score < 4 at 5 min, treated PDA, and all variables listed in this table.

## Discussion

This study identified several risk factors for TR-ROP in Japan from 2009 to 2018, including lower gestational age and birth weight, blood transfusion, prolonged invasive ventilation, sepsis, IVH, and delayed achievement of full enteral feeding; in contrast, the use of CPAP was protective against the development of TR-ROP. Prolonged oxygen exposure, which has been reported to be a major risk factor for ROP, was not an independent risk factor of ROP in this study.

To date, there have been few population-based reports examining the proportion of patients with and the risk factors of TR-ROP in the Asian population. In Taiwan, it was reported that the proportion of patients with TR-ROP was 6.5% (266/4096) among ROP patients born weighing ≤ 2000 g or born at a gestational age ≤ 32 weeks, based on a nationwide retrospective cross-sectional study from 2002 to 2011^20^. In another study from Taiwan, it has been reported that male sex, birth weight < 1250 g, RDS, and PDA are risk factors of TR-ROP; however, the variables used for multivariable analysis in this study are unknown due to the publication form of conference proceedings^21^. In Korea, it was reported that the proportion of patients with TR-ROP was 3.0% (1246/42,300) among all ROP patients and 25.0% (561/2240) among ROP patients born at < 28 weeks of gestation, based on a nationwide epidemiological study from 2007 to 2018^22^. The proportion of patients with TR-ROP in our study [(30.3% (4136/13,645)] was very close to the Japanese data reported by the iNeo study (30.4%)^23^, and was higher than that reported in the aforementioned Asian studies. Thus, it is likely that compared to other countries, ophthalmologists in Japan treat infants at an earlier stage of ROP^23^. Additionally, in recent times, anti-vasoendothelial growth factor (anti-VEGF) antibody treatment has become widespread in many facilities in Japan; this can be seen in the data captured throughout the length of this study (9.2% [380/4136 cases], Supplementary Fig. S4), and thus treatment for ROP may be increasing due to the convenience of this treatment.

In this study, we have clarified that gestational age < 26 weeks, blood transfusion, invasive ventilation ≥ 28 days, sepsis, and birth weight < 750 g are risk factors for TR-ROP, while the use of CPAP is a protective factor for TR-ROP in Japan. To date, only two large scale studies have investigated the risk factors for TR-ROP. Slidsborg et al., performed a retrospective register-based cohort study including 6490 preterm infants born at < 32 gestational weeks in Denmark from 1997 to 2008, and reported that the proportion of patients with TR-ROP was 4% (192/6490). In their study, gestational age at delivery, small for gestational age, male sex, mechanical ventilation, and blood transfusion were independent risk factors for TR-ROP among 31 known risk factors^24^. In the United States, Gonski et al., performed a database study involving 2306 TR-ROP infants out of 75,821 infants born weighing ≤ 1500 g or born at a gestational age ≤ 30 weeks, who were screened for ROP between 2006–2015; they reported that lower birth weight, mechanical ventilation on postnatal day 28, male sex, IVH, bacteremia, increased FiO_2_ on postnatal day 28, lack of antenatal steroids, white race, change in weight Z-score of ≥ 0, and NEC were independent risk factors for TR-ROP^25^. The independent risk factors for TR-ROP in our study (lower gestational age, lower birth weight, and duration of artificial ventilation) were generally consistent with those of two previous major reports^24,25^. In addition, blood transfusion was consistent with the former report and sepsis with the latter. The duration of CPAP was found to be an independent protective factor for TR-ROP in this study; this is a novel finding that has not been previously reported. Additionally, supplemental oxygen for ≥ 28 days, which has been reported as a major risk factor for ROP, was not a risk factor in this study. In high-income countries with adequate resources, where oxygen saturation is well controlled, oxygen administration is no longer reported as a risk for TR-ROP. However, multicenter studies from resource-limited countries such as Rwanda^26^ and China^27^ still suggest that oxygen administration is a risk factor for TR-ROP in low- and middle-income countries.

Regarding the ventilation strategy, Slidsborg et al., hypothesized that longer use of mechanical ventilation might worsen ROP through high-pressure oxygen and fluctuating oxygen levels^24^. Interestingly, in our study, a long duration of invasive ventilation was a risk factor for TR-ROP, while the duration of CPAP was protective. Similarly, a group at the University of Viena reported a higher rate of early CPAP treatment at birth (45–86% vs. 37–63%) and a lower proportion of patients with severe ROP (1–10% vs. 8–12%) than those of the Vermont Oxford Neonatal Network. They speculated that the lower rates of ROP in their institution might be related to the early initiation of CPAP^28^. However, the long-term advantage of CPAP has not been fully elucidated until now. A multicenter longitudinal follow-up study in Australia clarified that increased duration of assisted ventilation does not improve the rate of oxygen dependence at 36 weeks or lung function in childhood^29^. In addition, Nakashima et al., reported that the duration of CPAP use in NICUs in Japan has increased in recent years; however, the rate of bronchopulmonary dysplasia has increased in survivors between 2003 and 2016^30^. In Japan, mechanical ventilation is the most common respiratory strategy for preterm infants with respiratory distress within the first 48 h of life, which is clearly different from other countries that prioritize CPAP^31^.

The effect of CPAP on the development of TR-ROP has not been fully investigated in previous studies. In a study in Denmark, CPAP therapy was identified as a risk factor for TR-ROP in univariate analysis (OR 1.86 [1.17–3.31]); however, their study only divided the study population into two groups based on the presence or absence of CPAP use; they did not consider the duration of CPAP like we did in our study^24^. In a study from the United States, CPAP was not investigated as an independent variable but as part of respiratory support in univariate analysis, and the proportion of patients on CPAP support on day 28 was similar between the TR-ROP and non-TR-ROP groups (12% vs. 10%)^25^. Similarly, in a single-center study in Japan, use of CPAP at 35 weeks of postmenstrual age was reported as an independent factor for predicting TR-ROP among infants with ROP (OR 4.50)^32^. Unlike these previous reports, our study identified the duration of CPAP as a protective factor for TR-ROP. The main difference between our study and previous ones is that we examined the duration of CPAP as a continuous variable, while previous studies examined the presence or absence of CPAP use at specific time points as a nominal variable. A limitation of all risk-factor studies to date is that their data have not been analyzed using survival analysis, which takes into account both the baseline clinical characteristics of the newborns and the changes that occur while in the NICU. We believe that it is better to analyze the “duration” of CPAP rather than its “use at a specific timepoint”, and the duration of ventilation.

Intriguingly, the duration of ventilation and CPAP were independent risk and protective factors of TR-ROP, respectively. We hypothesize that CPAP may mediate its protective effect by two possible mechanisms. First, CPAP improves oxygenation by reducing lung damage and increasing functional residual capacity^33^; it stabilizes systemic oxygenation to prevent retinal hypoxia and avoid the need for high-concentration oxygen therapy. Second, since the frequency of desaturation within 8 weeks of gestation and that of severe ROP are significantly associated in preterm infants born at < 28 weeks gestation^34^, CPAP use post-extubation, which reduces apnea and desaturation events associated with fluctuations of oxygen saturation^35^, might protect from TR-ROP. Thus, we hypothesized that the proportion of patients with TR-ROP could be reduced if artificial ventilation can be successfully switched to CPAP.

There are several limitations in this study. First, due to the retrospective nature of the study, there is insufficient information on the stage and treatment of ROP and the details regarding sepsis, if it was early or late. Thus, we could not perform detailed analysis according to disease severity. Second, in Japan, there might be inter-facility differences in policies for ROP treatment (including the initial time of examination, frequency of examination, and the threshold for treatment) and for respiratory support strategies such as oxygen saturation targets and the time to switch from mechanical ventilation to CPAP. For ROP screening and treatment, it is desirable to use guidelines that are standardized across the globe. In addition, no data on noninvasive ventilation other than CPAP, such as high-flow oxygen therapy, was available despite the fact that the use of these treatments has increased in recent years. In addition, there is a possibility that infants with more severe RDS were ventilated while those in whom RDS was less severe were given CPAP; this might be a residual confounding factor in this retrospective study. Finally, the status of thrombocytopenia^16^, antenatal corticosteroid^17^, and failure to gain weight^36^, which are now considered as risk factors for ROP, should be analyzed in future studies. Therefore, the protective effect of CPAP use on the development of TR-ROP needs to be verified by future prospective studies, with a clear definition of TR-ROP.

In this nationwide survey, we found that lower gestational age and birth weight, blood transfusion, prolonged invasive ventilation, and sepsis were risk factors for TR-ROP; however, the use of CPAP was protective against the development of TR-ROP.

## Methods

### Patients and samples

This study was approved by the ethics committee of the Kobe University Graduate School of Medicine (approval number: B210042). All data were fully anonymized before they were accessed by any of the authors.

We retrospectively analyzed infants who were born alive between January 1, 2009, and December 31, 2018, and registered in the NRNJ, which is the same database as that used in the iNeo study. NRNJ prospectively registers the clinical information of all infants who are born weighing ≤ 1500 g or at a gestational age of < 32 weeks and admitted to the 218 participating NICUs (accounting for 53.8% of the 405 secondary- and tertiary-level NICUs in Japan, including Kobe University Hospital) as previously published^30^. In general, the data on each infant is entered in the database once, at discharge. The inclusion criteria were as follows: infants who were born between 22 and 27 weeks of gestation (< 28 weeks) and observed from birth to discharge in the same institute. We excluded infants who died before discharge, who had major anomalies or congenital diseases, who had been transferred to another hospital, and who had no available records regarding the diagnosis of ROP.

### Perinatal factors and TR-ROP

TR-ROP was defined as ROP requiring retinal laser photocoagulation and/or intravitreal anti-VEGF drugs^37^. The diagnosis and treatment of ROP was at the discretion of the attending ophthalmologist at each institution. The gestational age was calculated based on the results of an ultrasound examination in early pregnancy and the date of the last menstrual period. RDS was diagnosed by clinical and radiographic findings. Sepsis was defined as symptomatic culture-proven septicemia or bacteremia. Treated PDA was defined as PDA diagnosed based on both echocardiographic and clinical findings, and requiring medical or surgical treatment. NEC and IVH were both diagnosed by clinical and radiographic findings^38^. Invasive ventilation was defined as mechanical or high-frequency ventilation via an endotracheal tube, and CPAP included bilevel or continuous positive airway pressure equipped with a nasal mask or prongs according to previous publications^30^. Time to achieve full enteral feeding was defined as the number of days taken to first reach 100 ml/kg/day of enteral feeding. Parenteral nutrition was defined as use of intravenous hyperalimentation^39^.

### Statistical analyses

Data were described as median (IQR) or numbers (percentages). Missing data were excluded from the analysis as was done in similar bronchopulmonary dysplasia studies that used the same database^30^. The Mann–Whitney nonparametric rank, Fisher's exact, and Chi-square tests were used to compare the two groups. Chi-square test for trend or one-way analysis of variance test were used to assess the trend throughout the study period. Based on a previously published evidence^9^, a multivariable logistic regression analysis was performed to identify the factors associated with the development of TR-ROP. Associations among the following variables and TR-ROP were assessed: gestational age, birth weight, male sex, multiple births, Apgar score, RDS, sepsis, NEC, IVH, treated PDA, blood transfusion, erythropoietin for anemia, invasive ventilation, CPAP, supplemental oxygen, duration from birth to full enteral feeding, and parenteral nutrition. We included all variables in the regression model after transforming continuous variables into binary variables, including gestational age < 26 weeks, invasive ventilation ≥ 28 days, birth weight < 750 g, delayed achievement of full enteral feeding (> 14 days), supplemental oxygen ≥ 28 days, and CPAP ≥ 28 days. Adjusted values were obtained from a logistic regression analysis after adjusting for male sex, multiple births, Apgar score of < 4 points at 5 min, treated PDA, and all variables listed in Table 2. A *P* value of < 0.01 was considered statistically significant. Data were expressed as median (interquartile range), number (%), and odds ratio (99% confidence interval [CI]).

Statistical analyses were performed using JMP 15.0 (SAS Institute Inc., Cary, NC, USA), GraphPad Prism version 7.00 (GraphPad Software, La Jolla, CA, USA), and SPSS (version 22.0; IBM Corp., Armonk, NY, USA).

The study protocol was performed in accordance with the relevant guidelines and with the approval of the Ethics Committee at Kobe University Graduate School of Medicine (IRB approval number: B210042, 16/June/2021). Since this is a retrospective data analysis that does not use patient specimens and does not involve any physical contact with patients, we obtained permission from the committee and substituted individual consent with an opt-out statement on the website of Kobe University Hospital.

## Supplementary Information

Supplementary Figures.

## Data Availability

The data that support the findings of this study are available from NRNJ but restrictions apply to the availability of these data, which were used under license for the current study, and so are not publicly available. Data are, however, available from the authors upon reasonable request and with permission of NRNJ.
