# Supplementary material for: Long use of continuous positive airway pressure protects against the development of treatment-requiring retinopathy of prematurity
Source: Sci Rep. 2022 May 12;12:7799. doi: 10.1038/s41598-022-11509-w (PMC9098540; doi:10.1038/s41598-022-11509-w)
Supplement: Supplementary file 1 — Supplementary Figures. [file 41598_2022_11509_MOESM1_ESM.pptx]

## Slide 1
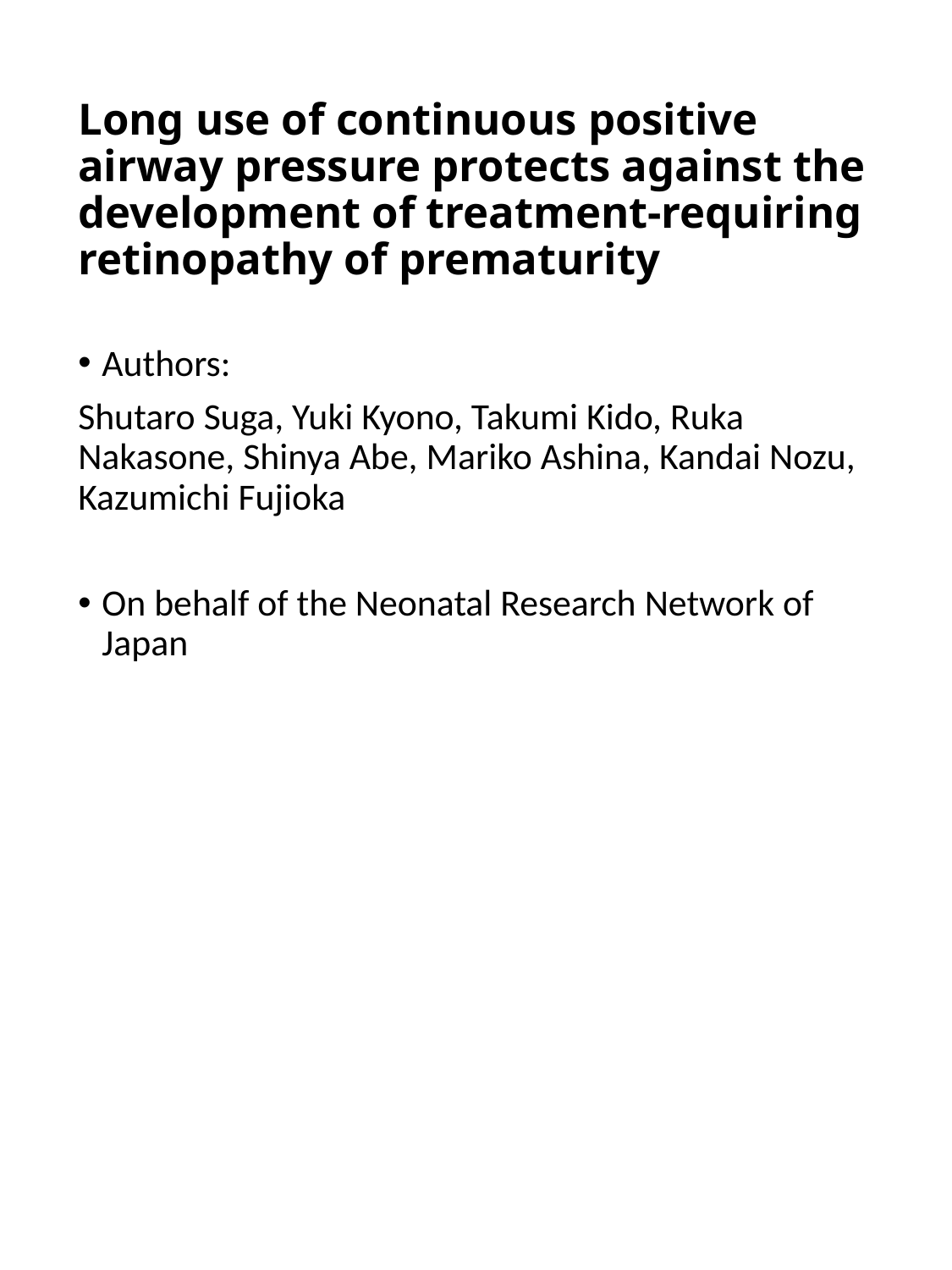

# Long use of continuous positive airway pressure protects against the development of treatment-requiring retinopathy of prematurity
Authors:
Shutaro Suga, Yuki Kyono, Takumi Kido, Ruka Nakasone, Shinya Abe, Mariko Ashina, Kandai Nozu, Kazumichi Fujioka
On behalf of the Neonatal Research Network of Japan

## Slide 2
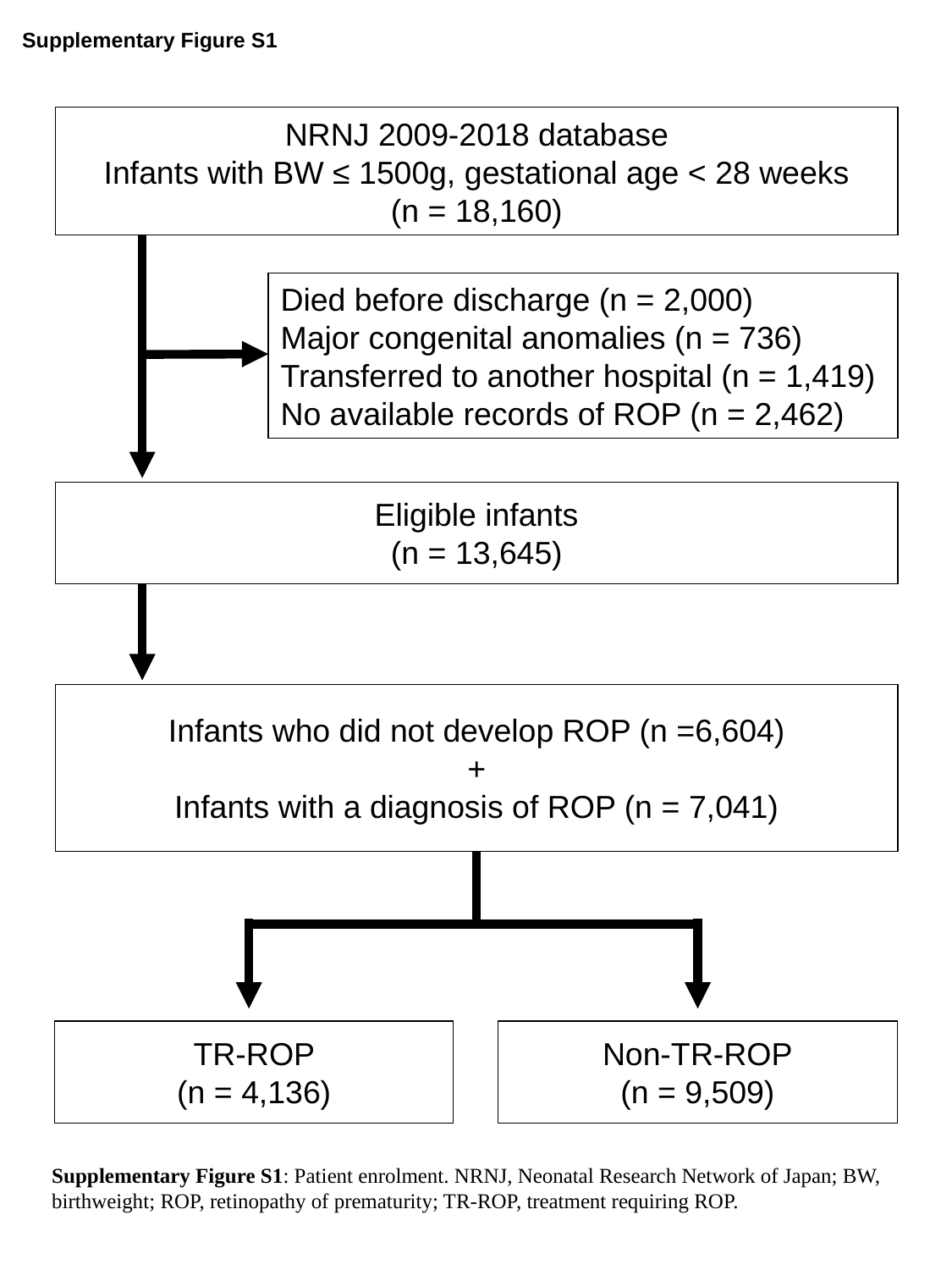

Supplementary Figure S1
NRNJ 2009-2018 database
Infants with BW ≤ 1500g, gestational age < 28 weeks
(n = 18,160)
Died before discharge (n = 2,000)
Major congenital anomalies (n = 736)
Transferred to another hospital (n = 1,419)
No available records of ROP (n = 2,462)
Eligible infants
(n = 13,645)
Infants who did not develop ROP (n =6,604)
+
Infants with a diagnosis of ROP (n = 7,041)
TR-ROP
(n = 4,136)
Non-TR-ROP
(n = 9,509)
Supplementary Figure S1: Patient enrolment. NRNJ, Neonatal Research Network of Japan; BW, birthweight; ROP, retinopathy of prematurity; TR-ROP, treatment requiring ROP.

## Slide 3
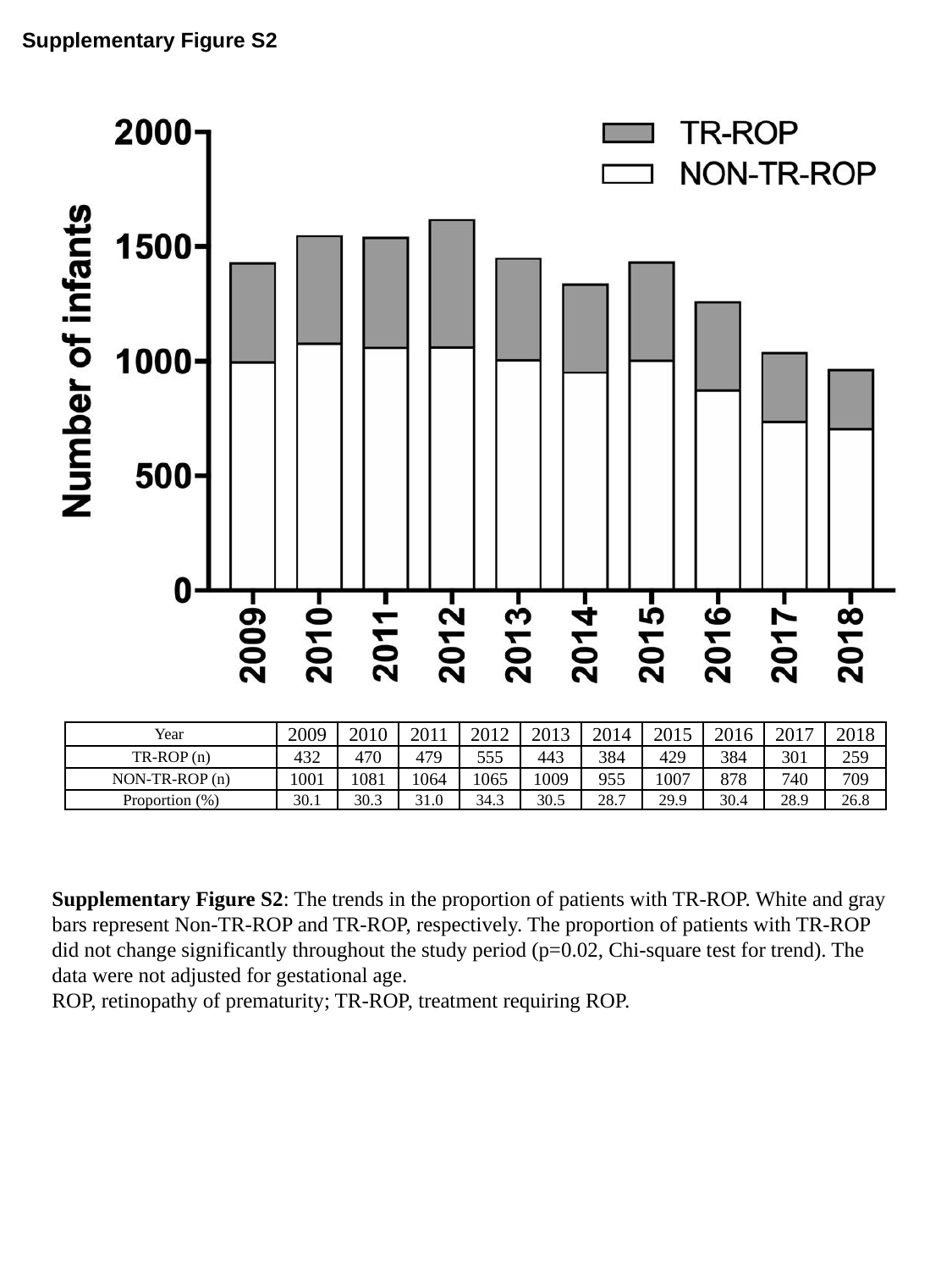

Supplementary Figure S2
| Year | 2009 | 2010 | 2011 | 2012 | 2013 | 2014 | 2015 | 2016 | 2017 | 2018 |
| --- | --- | --- | --- | --- | --- | --- | --- | --- | --- | --- |
| TR-ROP (n) | 432 | 470 | 479 | 555 | 443 | 384 | 429 | 384 | 301 | 259 |
| NON-TR-ROP (n) | 1001 | 1081 | 1064 | 1065 | 1009 | 955 | 1007 | 878 | 740 | 709 |
| Proportion (%) | 30.1 | 30.3 | 31.0 | 34.3 | 30.5 | 28.7 | 29.9 | 30.4 | 28.9 | 26.8 |
Supplementary Figure S2: The trends in the proportion of patients with TR-ROP. White and gray bars represent Non-TR-ROP and TR-ROP, respectively. The proportion of patients with TR-ROP did not change significantly throughout the study period (p=0.02, Chi-square test for trend). The data were not adjusted for gestational age.
ROP, retinopathy of prematurity; TR-ROP, treatment requiring ROP.

## Slide 4
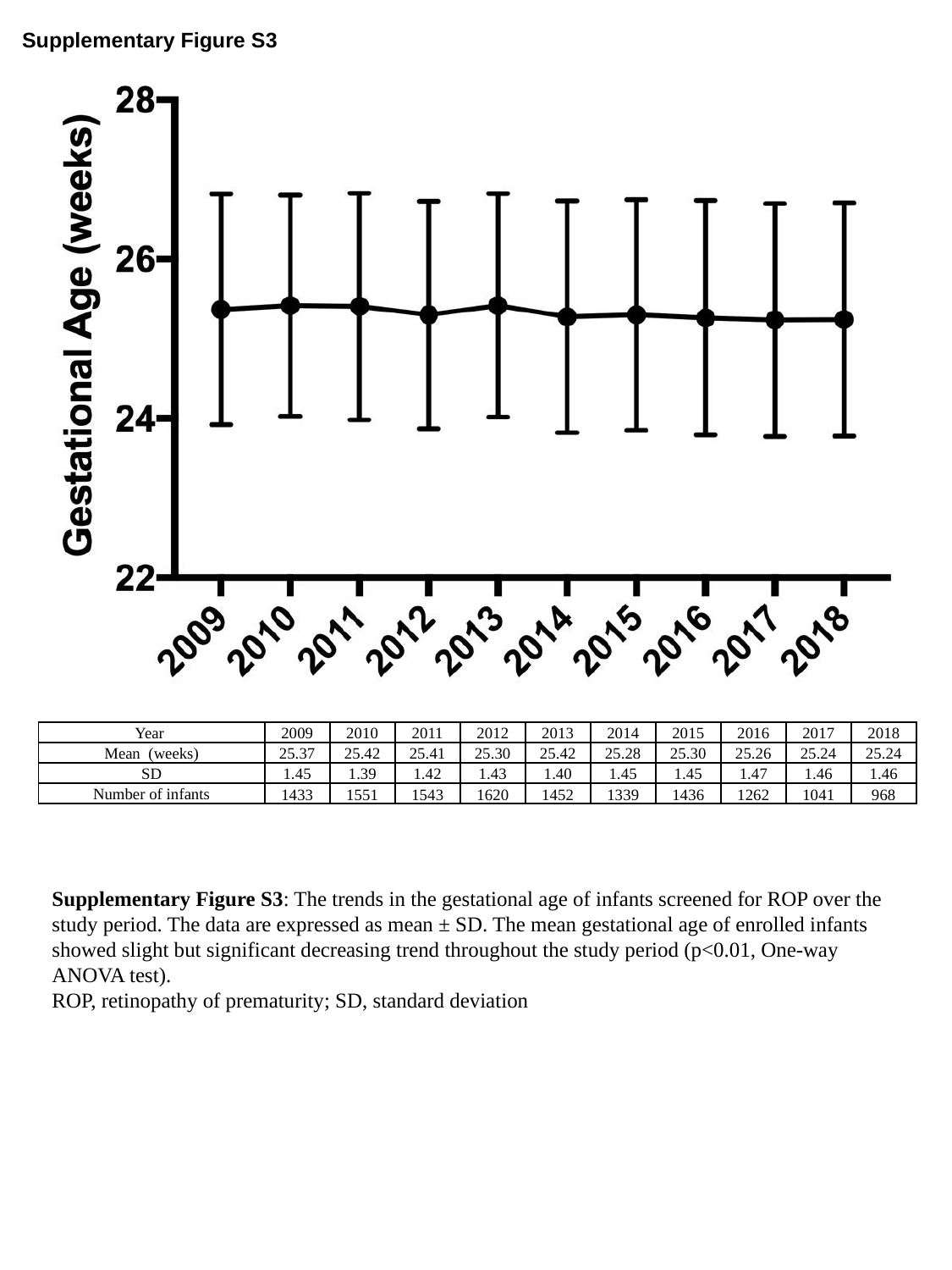

Supplementary Figure S3
| Year | 2009 | 2010 | 2011 | 2012 | 2013 | 2014 | 2015 | 2016 | 2017 | 2018 |
| --- | --- | --- | --- | --- | --- | --- | --- | --- | --- | --- |
| Mean (weeks) | 25.37 | 25.42 | 25.41 | 25.30 | 25.42 | 25.28 | 25.30 | 25.26 | 25.24 | 25.24 |
| SD | 1.45 | 1.39 | 1.42 | 1.43 | 1.40 | 1.45 | 1.45 | 1.47 | 1.46 | 1.46 |
| Number of infants | 1433 | 1551 | 1543 | 1620 | 1452 | 1339 | 1436 | 1262 | 1041 | 968 |
Supplementary Figure S3: The trends in the gestational age of infants screened for ROP over the study period. The data are expressed as mean ± SD. The mean gestational age of enrolled infants showed slight but significant decreasing trend throughout the study period (p<0.01, One-way ANOVA test).
ROP, retinopathy of prematurity; SD, standard deviation

## Slide 5
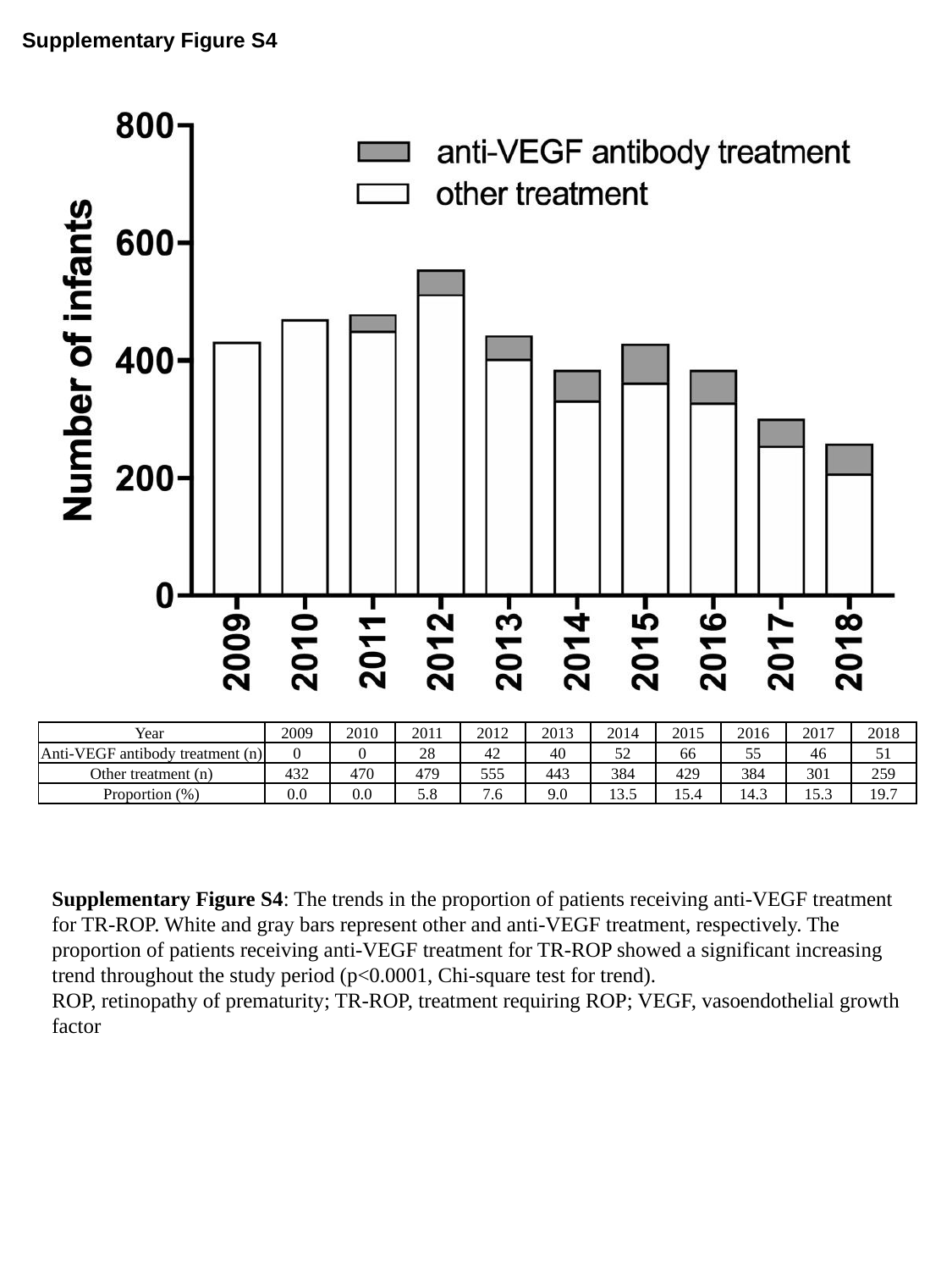

Supplementary Figure S4
| Year | 2009 | 2010 | 2011 | 2012 | 2013 | 2014 | 2015 | 2016 | 2017 | 2018 |
| --- | --- | --- | --- | --- | --- | --- | --- | --- | --- | --- |
| Anti-VEGF antibody treatment (n) | 0 | 0 | 28 | 42 | 40 | 52 | 66 | 55 | 46 | 51 |
| Other treatment (n) | 432 | 470 | 479 | 555 | 443 | 384 | 429 | 384 | 301 | 259 |
| Proportion (%) | 0.0 | 0.0 | 5.8 | 7.6 | 9.0 | 13.5 | 15.4 | 14.3 | 15.3 | 19.7 |
Supplementary Figure S4: The trends in the proportion of patients receiving anti-VEGF treatment for TR-ROP. White and gray bars represent other and anti-VEGF treatment, respectively. The proportion of patients receiving anti-VEGF treatment for TR-ROP showed a significant increasing trend throughout the study period (p<0.0001, Chi-square test for trend).
ROP, retinopathy of prematurity; TR-ROP, treatment requiring ROP; VEGF, vasoendothelial growth factor
